# Clinical Outcomes of Upfront Primary Tumor Resection in Synchronous Unresectable Metastatic Colorectal Cancer

**DOI:** 10.3390/cancers15205057

**Published:** 2023-10-19

**Authors:** Ji Eun Shin, Ho Jung An, Byoung Yong Shim, Hyunho Kim, Hyung Soon Park, Hyeon-Min Cho, Bong-Hyeon Kye, Ri Na Yoo, Ji-Yeon Moon, Sung Hwan Kim, Jonghoon Lee, Hyo Chun Lee, Ji-Han Jung, Kang-Moon Lee, Ji Min Lee

**Affiliations:** 1 Division of Oncology, Department of Internal Medicine, St. Vincent’s Hospital, College of Medicine, The Catholic University of Korea, Suwon 16247, Republic of Korea; wldms726@gmail.com (J.E.S.); shimby@catholic.ac.kr (B.Y.S.); h2kim@catholic.ac.kr (H.K.); happymeruk@catholic.ac.kr (H.S.P.); 2 Department of Surgery, St. Vincent’s Hospital, College of Medicine, The Catholic University of Korea, Suwon 16247, Republic of Korea; hmcho@catholic.ac.kr (H.-M.C.); ggbong@catholic.ac.kr (B.-H.K.); ninayoo1111@gmail.com (R.N.Y.); answl89@gmail.com (J.-Y.M.); 3 Department of Radiation Oncology, St. Vincent’s Hospital, College of Medicine, The Catholic University of Korea, Suwon 16247, Republic of Korea; kimandre@catholic.ac.kr (S.H.K.); koppul@catholic.ac.kr (J.L.); quepaso@catholic.ac.kr (H.C.L.); 4 Department of Hospital Pathology, St. Vincent’s Hospital, College of Medicine, The Catholic University of Korea, Suwon 16247, Republic of Korea; patholjjh7633@catholic.ac.kr; 5 Division of Gastroenterology, Department of Internal Medicine, St. Vincent’s Hospital, College of Medicine, The Catholic University of Korea, Suwon 16247, Republic of Korea; drmaloman@catholic.ac.kr (K.-M.L.); yulialee@naver.com (J.M.L.)

**Keywords:** colorectal cancer, primary tumor resection, synchronous, metastasis, asymptomatic

## Abstract

**Simple Summary:**

The role of upfront primary tumor resection (PTR) in patients with unresectable synchronous metastatic colorectal cancer without severe symptoms remains controversial. This study aimed to report the clinical outcomes of synchronous unresectable stage IV colorectal cancer patients with or without upfront PTR. A subgroup analysis was performed to determine clinical characteristics associated with better PTR outcomes. In this retrospective study, upfront PTR was not associated with overall survival (OS) after adjusting for other variables. Subgroup analysis revealed that the male sex, good performance, the T3 stage, the M1a stage, <2 organ metastases, and the administration of targeted agents, especially bevacizumab, seemed to be related to survival benefits after PTR. Upfront PTR could be considered in some subgroups, but these findings require larger studies to verify.

**Abstract:**

The role of upfront primary tumor resection (PTR) in patients with unresectable metastatic colorectal cancer without severe symptoms remains controversial. We retrospectively analyzed the role of PTR in overall survival (OS) in this population. Among the 205 patients who enrolled, the PTR group (n = 42) showed better performance (*p* = 0.061), had higher frequencies of right-sided origin (*p* = 0.058), the T4 stage (*p* = 0.003), the M1a stage (*p* = 0.012), and <2 organ metastases (*p* = 0.002), and received fewer targeted agents (*p* = 0.011) than the chemotherapy group (n = 163). The PTR group showed a trend for longer OS (20.5 versus 16.0 months, *p* = 0.064) but was not related to OS in Cox regression multivariate analysis (*p* = 0.220). The male sex (*p* = 0.061), a good performance status (*p* = 0.078), the T3 stage (*p* = 0.060), the M1a stage (*p* = 0.042), <2 organ metastases (*p* = 0.035), an RAS wild tumor (*p* = 0.054), and the administration of targeted agents (*p* = 0.037), especially bevacizumab (*p* = 0.067), seemed to be related to PTR benefits. Upfront PTR could be considered beneficial in some subgroups, but these findings require larger studies to verify.

## 1. Introduction

Systemic chemotherapy is the primary treatment for patients with synchronous stage IV colorectal cancer (CRC). Over the past 20 years, advances in systemic treatments, including biologically targeted agents, have led to dramatic improvements in the overall survival (OS) of patients with stage IV CRC, exceeding 30 months [1]. Primary tumor resection (PTR) has been performed to manage tumor-related symptoms such as obstructions, perforations, and refractory bleeding in these populations. However, the role of upfront PTR in asymptomatic and mildly symptomatic patients remains controversial. Upfront PTR may prevent primary-tumor-related complications during the course of treatment, resulting in emergent surgery and poor oncological outcomes [2,3]. It can improve prognosis by removing the primary tumor source and reducing tumor-derived cytokines or chemokines [4]. However, this delays the administration of systemic treatment, and surgery-related complications are concerning [5,6]. 

Several retrospective, prospective cohorts or nationwide registry analyses have shown the survival benefit of the upfront PTR in unresectable metastatic CRC [7,8,9,10,11,12,13,14,15,16]. Yet the heterogeneity of the study population, systemic treatment, and inevitable selection bias prevented definitive conclusions. Furthermore, many variables associated with prognosis or clinical outcomes were missing [14,16]. Recent randomized prospective clinical trials have reported that the upfront PTR group did not show a survival benefit or increased 60-day mortality compared with the chemotherapy-first group [17,18,19]. However, most studies closed early owing to poor accrual or futility, and a substantial number of participants did not receive any treatment after randomization. 

In this study, we aimed to report the clinical outcomes of synchronous unresectable stage IV CRC patients with or without upfront PTR. A subgroup analysis was performed to determine clinical characteristics associated with better PTR outcomes.

## 2. Materials and Methods

### 2.1. Ethics Statements

This study was approved by the Institutional Review Board of St. Vincent Hospital (number: VC23RISI0179).

### 2.2. Study Design and Patients

We retrospectively reviewed the hospital database to identify all patients diagnosed with synchronous stage IV CRC between 2010 and 2020. The inclusion criteria were an age of at least 18 years, an initial diagnosis of unresectable stage IV colorectal adenocarcinoma according to TNM 8th edition [20], primary tumors without severe symptoms, and the receipt of systemic anti-cancer treatment. Severe primary tumor symptoms were defined as follows: perforation, fistula formation, bleeding causing hemodynamic instability, or obstruction not relieved by a noninvasive procedure.

### 2.3. Treatment and Assessment

PTR was performed in the same manner as the surgery for non-metastatic CRC, including an adequate level of lymphadenectomy. For chemotherapy, 5-fluorouracil-based cytotoxic agents were selected. Irinotecan or oxaliplatin was chosen as a combination partner and switched to the other way around when progressed if appropriate. Bevacizumab has been added since 2014, and cetuximab has been added for the population with wild *RAS* since 2015. Patients were assessed at 6–8-week intervals using computed tomography of the abdomen and chest and serum carcinoembryonic antigen (CEA) levels. If the tumor became resectable during the course of treatment in both groups, conversion to complete the resection of all metastatic sites and/or primary tumors was performed. Patients’ performance status at the time of treatment was determined using the Eastern Cooperative Oncology Group (ECOG) performance status scale [21].

### 2.4. Statistical Analysis

Categorical variables are presented as numbers and percentages and were compared using the chi-square or Fisher’s exact test. Continuous variables were expressed as median values (ranges) and were compared using Student’s unpaired *t*-test or the Mann–Whitney U test, as appropriate. A subgroup analysis was performed to determine clinical characteristics associated with better PTR outcomes. OS was measured from the date of the initial treatment (PTR or systemic treatment) until death due to any cause or the last censored date during follow-up. OS was calculated using the Kaplan–Meier method, and differences in survival between the groups were compared using the log-rank test. Cox proportional hazard regression methods were used to find the association between variables and survival. Variables with significance as defined by *p* < 0.30 in the univariate model were included in the multivariate model. Adjusted hazard ratios (HRs) with 95% confidence intervals (CIs) were calculated. Propensity score matching (PSM) analyses were performed to adjust for heterogeneity between two groups [22]. Multivariable logistic regression was used to generate a propensity score, predicting the treatment based on variables including ECOG performance status, the primary tumor location, the clinical T, the M stage, and the No. of organ metastases. Each patient then was assigned an estimated propensity score and matched 1:1 between the upfront PTR and upfront chemotherapy groups.

Cases with missing values were deleted listwise. A *p*-value < 0.05 was considered statistically significant. R version 4.2.2 was used to perform all statistical analyses (The R Foundation for Statistical Computing, Vienna, Austria; http://www.r-project.org/, accessed on 1 July 2022).

## 3. Results

### 3.1. Baseline Characteristics

**Figure 1:**
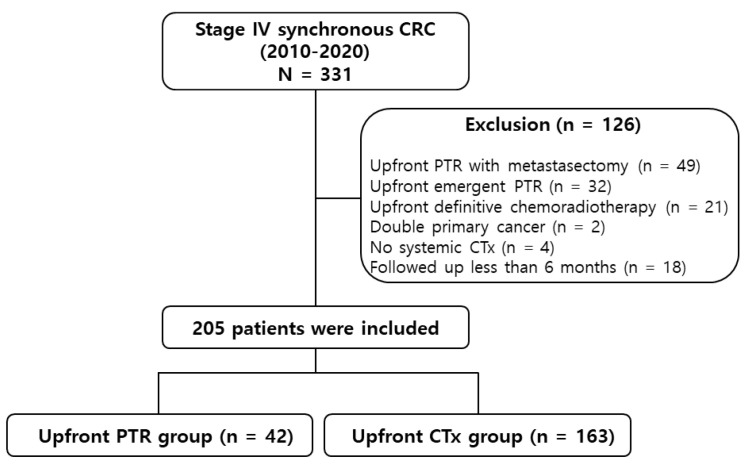
Patient selection flowchart. CRC: colorectal cancer; PTR: primary tumor resection; CTx: chemotherapy.

Among the 331 patients screened, 108 were excluded for the following reasons: 49 patients received initial metastasectomy for resectable metastases; 32 patients required emergent primary tumor resection due to severe symptoms; 21 patients underwent upfront long-course chemoradiotherapy; 2 patients had double primary malignancies along with CRC; 4 patients did not receive any systemic chemotherapy; and 18 patients were followed up with for less than 6 months. Finally, 205 patients were included in this analysis (Figure 1). Forty-two (20.5%) patients were treated with upfront PTR, and 163 (79.5%) were treated with upfront chemotherapy. The baseline patient characteristics are presented in Table 1. The two treatment groups were well balanced in terms of median age, sex, serum CEA level, tumor differentiation, clinical N stage, and *RAS* status. The median follow-up period was 18.0 months (range: 6.0–92.0 months). Patients who underwent upfront PTR showed a trend toward or significantly better ECOG performance status (0/1, *p* = 0.061), higher frequencies of right-sided colon cancer (*p* = 0.058), the T4 stage (*p* = 0.003), and the M1a stage (*p* = 0.012), and a lower number of organ metastases (0/1, *p* = 0.002). 

The upfront chemotherapy group received more irinotecan-based doublets (*p* < 0.001) and targeted agents (*p* = 0.011) than the upfront PTR group as the first line of systemic treatment. The mean times to start systemic treatment were 50.4 (±35.4) days in the upfront PTR group and 17.3 (±19.5) days in the upfront chemotherapy group (*p* < 0.001). The types of PTR and complications in the upfront PTR group are summarized in Table 2. During the course of chemotherapy, conversion to the complete resection of all metastatic sites resulting in a disease-free status was performed more frequently in the upfront PTR group than in the upfront chemotherapy group (21.4% vs. 12.9%, *p* = 0.006).

### 3.2. Variables Associated with OS

**Figure 2:**
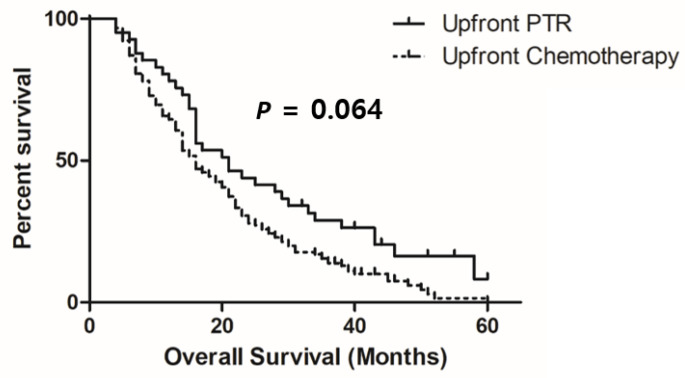
A Kaplan–Meier curve of overall survival. PTR: primary tumor resection.

The median OS was 16.0 months (range: 6–92.0 months) in the whole study population. Kaplan–Meier curves showed that the upfront PTR group had a longer OS than the upfront chemotherapy group (20.5 versus (vs.) 16.0 months, *p* = 0.064; Figure 2). In univariate Cox regression analysis, OS was associated with age, ECOG performance status, primary tumor location, serum CEA level, tumor differentiation, the administration of targeted agents, and upfront PTR (Table 3). Multivariate analysis showed that old age (HR: 1.577; 95% CI, 1.125–2.212; *p* = 0.008), right-side colon cancer (HR: 1.503; 95% CI, 1.057–2.136; *p* = 0.023), a high CEA level (HR: 1.407; 95% CI, 1.006–1.967; *p* = 0.046), and poor differentiation (HR: 2.476; 95% CI, 1.327–4.618; *p* = 0.004) were associated with poorer OS, whereas the administration of a targeted agent was associated with a longer OS (HR: 0.582; 95% CI, 0.383–0.885; *p* = 0.011). Upfront PTR was not significantly associated with OS in multivariate analysis (HR: 0.763; 95% CI, 0.496–1.175; *p* = 0.220). Patients receiving upfront PTR or chemotherapy did not show any survival differences after PSM matching (*p* = 0.220, Table S1 and Figure S1).

### 3.3. Subgroup Analysis Favored Upfront PTR 

**Figure 3:**
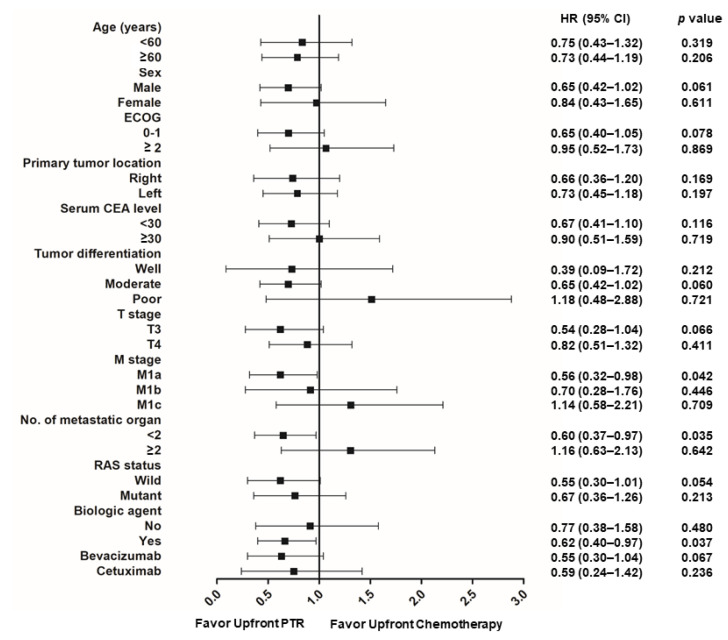
Forest plot of overall survival. PTR: primary tumor resection; ECOG: Eastern Cooperative Oncology Group; CEA: carcinoembryonic antigen; CI: confidence interval; HR: hazard ratio.

Subgroup analyses were performed to identify the clinical subgroups that benefited from upfront PTR (Figure 3). The male sex (*p* = 0.061), a good performance status (*p* = 0.078), the T3 stage (*p* = 0.066), the M1a stage (*p* = 0.042), <2 organ metastases (*p* = 0.035), and an RAS wide tumor (*p* = 0.054) showed a trend toward longer OS when upfront PTR was performed. Upfront PTR was associated with longer OS in patients who received targeted agents (*p* = 0.037), especially in those treated with bevacizumab (*p* = 0.067).

### 3.4. Primary-Tumor-Related Complications in the Upfront Chemotherapy Group during Treatment

In the upfront chemotherapy group, 45 (27.6%) patients experienced primary-tumor-related complications, including obstructions (18.4%), bleeding (3.1%), pain (1.8%), perforations (1.2%), fistulas (1.2%), abscesses (1.2%), and ischemic changes (0.6%). There was no significant difference between the right- and left-sided tumors. Twenty-five patients received surgical treatment, 14 patients were relieved via non-surgical treatment, and five patients did not recover and died. The median survival time after complications was 83 (1–1, 321) days. 

## 4. Discussion

In this study, about 20% of synchronous metastatic CRC patients received upfront PTR with no or few primary tumor symptoms. This indicates that PTR was performed in highly selected patients, and most patients received chemotherapy as an initial treatment. The PTR group showed a trend for longer OS in univariate analysis, but this was not statistically significant after adjusting for other variables. PTR seemed to be beneficial in some subgroups: male patients and patients with a good performance status, T3 or M1a stage, <2 organ metastases, an RAS wild tumor, and the administration of a targeted agent, especially bevacizumab. Primary-tumor-related complications occurred in 27.6% of patients in the upfront chemotherapy group, but most were relieved via surgery or intervention.

The upfront PTR for patients with initial stage IV CRC without severe primary tumor symptoms was performed at various frequencies according to the surgeon’s discretion or multidisciplinary team policy. The overall frequency of PTR has decreased recently [23,24,25], but many clinicians continue to perform upfront PTR before chemotherapy to prevent primary-tumor-related complications during the course of treatment and/or to improve OS. 

The primary-tumor-related complication rate in patients with CRC receiving chemotherapy varies between 11% and 35%, and approximately half of the patients require surgical intervention [5,16,26,27,28,29,30,31]. Here, a quarter of patients in the upfront chemotherapy group experienced primary-tumor-related complications. Most complications were relieved via surgery or intervention; however, a few patients did not recover because the complications occurred near the end of life. Obstruction was the most common complication, which is consistent with other studies’ findings [5,31]. Emergent colectomy is associated with higher morbidity and mortality rates than elective surgery [2,3]. In this study, colectomy was performed in only 14 patients, whereas the other patients were treated with bypasses, stent insertions, and radiation. Patients could be divided into three groups: chemotherapy only, chemotherapy followed by secondary PTR due to complications, and upfront PTR followed by chemotherapy. Survival analysis revealed that upfront or secondary PTR did not seem to show survival differences. This could be interpreted as indicating that PTR could be performed during the course of chemotherapy if it is needed.

In the era of modern chemotherapy and targeted agents, OS, tumor response, and disease control rates have increased. The frequency of primary-tumor-related complications since 2000 has continued to vary; therefore, it is not clear how they have changed since modern systemic treatments have been introduced [6,26,27,28,29,30,32]. Furthermore, there are concerns about the use of bevacizumab when the primary tumor is not resected because bevacizumab can cause bleeding, a fistula, or bowel perforation. The effect of bevacizumab administration on the PTR benefit is still controversial [7,26,28,32]. Some studies have reported that upfront PTR is associated with longer OS in bevacizumab-treated patients with CRC [26,33]. In this study, a higher rate of primary-tumor-related complications was observed in the subgroups treated with targeted agents; however, the difference was not statistically significant (22.7% vs. 12.2%, *p* = 0.136), which could be due to the longer OS in this population. The frequency did not differ according to the type of targeted agent used (23.5% for bevacizumab and 20.0% for cetuximab). Yet, surgical treatments were performed more frequently in the bevacizumab-treated subgroup than in the non-bevacizumab treatment subgroup (13.7% vs. 7.9%), which could partially explain why upfront PTR seemed to be favored in the bevacizumab subgroup in the subgroup analysis.

The survival benefit of upfront PTR in patients with synchronous metastatic CRC has only been demonstrated in retrospectively analyzed studies. Selection bias was inevitable in cases in which upfront PTR was performed: those with a good performance status, liver-only metastasis, few organ metastases, a non-rectal origin, or low serum CEA levels [10,15,34,35,36,37,38,39,40,41], which could have misleading results. Additionally, the study population was heterogeneous in terms of the presence of symptoms, the timing of PTR (before or during chemotherapy), and/or the application or type of systemic treatment [7,8,9,10,11,12,13,15,35,42]. To adjust for these imbalances and heterogeneity, some studies have applied statistical methods, such as multivariate analysis or propensity matching [25,42,43]. Several studies, including ours, have shown that PTR is not associated with improved OS after adjusting for confounding factors [6,25,42,44]. Moreover, prognostic variables and therapeutic strategies have evolved over the decades, and insufficient data collection in many studies makes the role of upfront PTR debatable [14,16].

The role of the PTR in OS remains controversial in the era of biologic-targeted agents [26,42,45,46]. A few large, prospective, randomized trials comparing upfront PTR with bevacizumab plus chemotherapy have been conducted to answer this question and concluded that upfront PTR was futile in terms of 60-day mortality or OS. However, most were closed early due to poor accrual or the assumed futility of the upfront PTR, which limited the statistical power supporting the conclusions [17,18]. iPACS was the first randomized controlled trial to suggest the utility of upfront PTR for asymptomatic, synchronous, unresectable metastatic CRC. However, it enrolled patients with ≤3 metastatic diseases, and more than half were T3 or N0/1, which could question true unresectability [17]. Rahbari et al. also reported that upfront PTR did not prolong OS; however, more patients in the PTR group did not receive any systemic treatment after PTR, similar to the iPACS study [19]. This advantage of PTR for asymptomatic patients with CRC is difficult to validate in randomized clinical trials because many factors are involved in the decision-making process of PTR, including patient or clinician preference and various clinical situations that cannot be easily controlled in clinical trials. The results of randomized clinical trials are summarized in Table S2. 

Subgroup analyses could provide clues as to which patients could benefit from PTR. First, PTR could be associated with improved OS when performed in patients with a good performance status who can receive systemic treatment after PTR. The administration of polychemotherapy is a key determinant of OS [7]. In particular, patients receiving targeted agents showed a significantly favorable prognosis after PTR compared with the subgroup receiving chemotherapy alone. This indicates that patients with good performance who can tolerate and are willing to receive active systemic treatment could consider upfront PTR to improve their OS. The PTR group showed a considerable delay in chemotherapy administration; therefore, PTR should be avoided in patients whose conditions can rapidly deteriorate. 

The extent of metastasis is also an important factor. Our study showed that patients with less extensive organ metastasis had favorable outcomes after PTR, which is consistent with other studies’ results [37,47,48]. The serum CEA level reflects the extent of the tumor burden, and the subgroup with a low CEA level was also associated with PTR benefit [38]. The benefits of PTR on OS could differ according to the primary tumor site. In our study, upfront PTR was performed more frequently in patients with right-sided colon cancer. It could be assumed that upfront PTR was performed for cancer diagnosis purposes or to prevent future severe symptoms like bleeding or obstructions in right-side colon cancer. For left-side colon cancer, diagnostic or therapeutic procedures could be easily performed, which explains the dominance of right-sided colon cancer in the upfront PTR group. Several studies have reported that right-sided colon cancer is related to a reduced OS benefit after PTR compared to left-sided tumors [18,49,50]. Right-sided colon cancer is associated with poorly differentiated histology, an advanced stage at diagnosis, *BRAF* mutations, or consensus molecular subtype 1, which is related to a poor prognosis. In this study, PTR benefits did not differ according to primary sites. Only a few studies have reported controversial results regarding the presence of tumor *RAS* mutations [33,51], which were related to the PTR benefits in this study. Age did not affect the benefits of PTR [24,52], whereas the female sex showed more upfront PTR benefits than the male sex, including in this study [7].

This study had some limitations. First, this study was conducted at a single center, and the sample size was too small, especially in the PTR group, to draw statistically significant results. Second, some data were inaccurate or missing due to the retrospective design of the study. We could not clarify the exact reasons for upfront PTR. However, we assumed that upfront PTR might have been performed to prevent future complications like obstructions, bleeding, pain, or fistula formation even though these were not severe at the time of diagnosis, which could be supported by the fact that there were more right-sided and T4 stage cancers in the PTR group (Table 1). Another reason might be surgeons’ opinion that upfront PTR could improve overall prognosis, especially in patients with good performance and lower tumor burdens. Third, our study enrolled patients between 2010 and 2020; standard chemotherapy has changed, and some patients did not have molecular results associated with clinical outcomes. Since the mid-2010s, biologic agents, including anti-EGFR and anti-VEGF, have been widely used. Recently, Her2 or BRAF inhibitors, immunotherapy, or modern liver-directed local therapy have been increasingly used in metastatic CRC. Few patients received these treatments (n = 18; Her2 inhibitor (n = 3), BRAF inhibitor (n = 2), immunotherapy for microsatellite instability (n = 1), liver radiofrequency ablation (n = 2), and stereotactic body radiation therapy (n = 12)). The clinical significance of upfront PTR did not differ according to the administration of modern treatments. Finally, all patients received systemic therapy, which does not reflect the fact that some did not receive further treatment with or without PTR. Nonetheless, we attempted to collect variables associated with clinical outcomes in patients with stage IV CRC and showed the clinical role of PTR on OS according to these variables. 

## 5. Conclusions

For asymptomatic or mild symptomatic stage IV CRC patients, systemic chemotherapy, including biological agents, is the main treatment. Upfront PTR could be considered beneficial in some subgroups. Further large prospective trials are needed to validate our results.

## Figures and Tables

**Table 1:** Baseline characteristics.

Characteristic	Upfront PTR	Upfront Chemotherapy	*p*-Value
	N = 42 (%)	n = 163 (%)	
Age (years)			
Median (range)	60 (34–84)	63 (30–82)	0.290
Sex			
Male	31 (73.8)	103 (63.2)	0.197
Female	11 (26.2)	60 (36.8)	
ECOG performance status			
0/1	29 (69.0)	89 (54.6)	0.091
≥2	13 (31.0)	74 (45.4)	
Primary tumor location			
Right-sided	17 (40.5)	43 (26.4)	0.058
Left-sided	25 (59.5)	120 (73.6)	
CEA (ng/mL)	13.3 (1.0–594)	33.0 (0–86,002)	0.480
Tumor differentiation			
Well	4 (9.5)	20 (12.3)	0.666
Moderate	31 (73.8)	124 (76.1)	
Poor	7 (16.7)	19 (11.6)	
Clinical T stage			
T3	13 (31.0)	92 (56.4)	0.003
T4	29 (69.0)	71 (43.6)	
Clinical N stage			
N0	2 (4.8)	6 (3.7)	0.864
N1	10 (23.8)	45 (27.6)	
N2	30 (71.4)	112 (68.7)	
Clinical M stage			
M1a	21 (50.0)	69 (42.3)	0.012
M1b	6 (14.3)	60 (36.8)	
M1c	15 (35.7)	34 (20.9)	
No. of organ metastases			
0 or 1	30 (71.4)	74 (45.4)	0.003
≥2	12 (28.6)	89 (54.6)	
Liver metastases	20 (47.6)	125 (76.7)	<0.001
Median number	2 (1–20)	10 (1–21)	<0.001
Maximal size (cm)	2.0 (0.8–7.5)	4.4 (0.3–17.0)	<0.001
RAS status			
Wild	16 (38.1)	81 (49.7)	0.723
Mutant	15 (35.7)	66 (40.5)	
NA	11 (26.2)	16 (19.8)	
Time to chemotherapy (days)	50.4 (±35.4)	17.8 (±19.5)	<0.001
First-line chemotherapy			
Fluoropyrimidine alone	5 (11.9)	1 (0.6)	<0.001
Irinotecan doublet	15 (35.7)	101 (62.0)	
Oxaliplatin doublet	22 (52.4)	61 (37.4)	
First-line targeted gent			
Cetuximab	9 (21.4)	73 (44.8)	<0.001
Bevacizumab	13 (31.0)	62(38.0)	
No	20 (47.6)	28 (17.2)	
Administration of targeted agent	29 (69.0)	140 (85.9)	0.011
No. of lines of systemic treatment	2 (1–7)	2 (1–7)	0.811
Conversion to complete tumor resection	9 (21.4) *	21 (12.9) **	0.006

PTR: primary tumor resection; ECOG: Eastern Cooperative Oncology Group; CEA: carcinoembryonic antigen; NA: not available. * The complete resection of all metastasis sites during chemotherapy; ** The complete resection of the primary site and all metastatic sites during chemotherapy.

**Table 2:** Operative features in the upfront PTR group (n = 42).

Type of Operation	Number (%)
Anterior resection	16
With small bowel segmental resection	1
Low anterior resection	2
With T-loop colostomy	1
With loop ileostomy	1
Hartmann’s operation	1
Segmental resection of the descending colon	1
Left hemicolectomy	2
Right hemicolectomy	14
With duodenal resection	1
With small bowel segmental resection	1
Subtotal colectomy	1
**Postoperative clinical outcomes**	
Hospital stay (days)	14.4 ± 6.2 (9–34)
Complications	7 (16.7)
Ileus	6 (14.3)
Intraabdominal abscess	1 (2.4)
Mortality	0

**Table 3:** Variables associated with overall survival.

	Univariate	Multivariate	
	*p*-Value	HR (95% CI)	*p*-Value
Age (≥60)	0.003	1.577 (1.125–2.212)	0.008
Male sex	0.270	1.206 (0.866–1.681)	0.268
ECOG ≥ 2	0.013	1.250 (0.911–1.715)	0.167
Right-side colon cancer	0.018	1.503 (10057–2.136)	0.023
CEA ≥ 30 (ng/mL)	0.066	1.407 (1.006–1.967)	0.046
Tumor differentiation	0.006	2.476 (1.327–4.618)	0.004
Clinical T4 stage	0.990		
Clinical N1/2 stage	0.309		
Clinical M1c stage	0.148	1.505 (0.990–2.289)	0.056
No. of organ metastases (≥2)	0.149	1.322 (0.939–1.861)	0.109
Liver metastasis	0.691		
RAS mutation	0.802		
Administration of a targeted agent	0.009	0.582 (0.383–0.885)	0.011
Upfront PTR	0.053	0.763 (0.496–1.175)	0.220

HR: hazard ratio; CI: confidence interval; ECOG: Eastern Cooperative Oncology Group; CEA: carcinoembryonic antigen; PTR: primary tumor resection.

## Data Availability

The data are available upon request from the corresponding author.

## Supplementary Materials

The following supporting information can be downloaded at: https://www.mdpi.com/article/10.3390/cancers15205057/s1. Table S1: Propensity score-matched pairs (n = 84); Figure S1: A Kaplan–Meier curve of overall survival in propensity matched population (n = 84). PTR, primary tumor resection. Table S2. Summary of randomized trials comparing upfront PTR and CTx.

## References

[1] Venook A.P., Niedzwiecki D., Lenz H.-J., Innocenti F., Fruth B., Meyerhardt J.A., Schrag D., Greene C., O’Neil B.H., Atkins J.N. (2017). Effect of First-Line Chemotherapy Combined with Cetuximab or Bevacizumab on Overall Survival in Patients with KRAS Wild-Type Advanced or Metastatic Colorectal Cancer: A Randomized Clinical Trial. JAMA.

[2] Mun J.Y., Kim J.E., Yoo N., Cho H.M., Kim H., An H.J., Kye B.H. (2022). Survival Outcomes after Elective or Emergency Surgery for Synchronous Stage IV Colorectal Cancer. Biomedicines.

[3] Xu Z., Becerra A.Z., Aquina C.T., Hensley B.J., Justiniano C.F., Boodry C., Swanger A.A., Arsalanizadeh R., Noyes K., Monson J.R. (2017). Emergent Colectomy Is Independently Associated with Decreased Long-Term Overall Survival in Colon Cancer Patients. J. Gastrointest. Surg..

[4] Kim M.Y., Oskarsson T., Acharyya S., Nguyen D.X., Zhang X.H., Norton L., Massagué J. (2009). Tumor self-seeding by circulating cancer cells. Cell.

[5] Scheer M.G., Sloots C.E., van der Wilt G.J., Ruers T.J. (2008). Management of patients with asymptomatic colorectal cancer and synchronous irresectable metastases. Ann. Oncol..

[6] Niitsu H., Hinoi T., Shimomura M., Egi H., Hattori M., Ishizaki Y., Adachi T., Saito Y., Miguchi M., Sawada H. (2015). Up-front systemic chemotherapy is a feasible option compared to primary tumor resection followed by chemotherapy for colorectal cancer with unresectable synchronous metastases. World J. Surg. Oncol..

[7] Colloca G.A., Venturino A., Guarneri D. (2022). Primary tumor resection in patients with unresectable colorectal cancer with synchronous metastases could improve the activity of poly-chemotherapy: A trial-level meta-analysis. Surg. Oncol..

[8] Stillwell A.P., Buettner P.G., Ho Y.H. (2010). Meta-analysis of survival of patients with stage IV colorectal cancer managed with surgical resection versus chemotherapy alone. World J. Surg..

[9] Anwar S., Peter M.B., Dent J., Scott N.A. (2012). Palliative excisional surgery for primary colorectal cancer in patients with incurable metastatic disease. Is there a survival benefit? A systematic review. Colorectal. Dis..

[10] Clancy C., Burke J.P., Barry M., Kalady M.F., Calvin Coffey J. (2014). A meta-analysis to determine the effect of primary tumor resection for stage IV colorectal cancer with unresectable metastases on patient survival. Ann. Surg. Oncol..

[11] Tarantino I., Warschkow R., Worni M., Cerny T., Ulrich A., Schmied B.M., Güller U. (2015). Prognostic Relevance of Palliative Primary Tumor Removal in 37,793 Metastatic Colorectal Cancer Patients: A Population-Based, Propensity Score-Adjusted Trend Analysis. Ann. Surg..

[12] Lee K.C., Ou Y.C., Hu W.H., Liu C.C., Chen H.H. (2016). Meta-analysis of outcomes of patients with stage IV colorectal cancer managed with chemotherapy/radiochemotherapy with and without primary tumor resection. Onco Targets Ther..

[13] Ha G.W., Kim J.H., Lee M.R. (2018). Meta-analysis of oncologic effect of primary tumor resection in patients with unresectable stage IV colorectal cancer in the era of modern systemic chemotherapy. Ann. Surg. Treat. Res..

[14] Harji D.P., Vallance A., Selgimann J., Bach S., Mohamed F., Brown J., Fearnhead N. (2018). A systematic analysis highlighting deficiencies in reported outcomes for patients with stage IV colorectal cancer undergoing palliative resection of the primary tumour. Eur. J. Surg. Oncol..

[15] Nitsche U., Stöß C., Stecher L., Wilhelm D., Friess H., Ceyhan G.O. (2018). Meta-analysis of outcomes following resection of the primary tumour in patients presenting with metastatic colorectal cancer. Br. J. Surg..

[16] Sterpetti A.V., Costi U., D’Ermo G. (2020). National statistics about resection of the primary tumor in asymptomatic patients with Stage IV colorectal cancer and unresectable metastases. Need for improvement in data collection. A systematic review with meta-analysis. Surg. Oncol..

[17] Kanemitsu Y., Shitara K., Mizusawa J., Hamaguchi T., Shida D., Komori K., Ikeda S., Ojima H., Ike H., Shiomi A. (2021). Primary Tumor Resection Plus Chemotherapy versus Chemotherapy Alone for Colorectal Cancer Patients with Asymptomatic, Synchronous Unresectable Metastases (JCOG1007; iPACS): A Randomized Clinical Trial. J. Clin. Oncol..

[18] van der Kruijssen D.E.W., Elias S.G., Vink G.R., van Rooijen K.L., Lam-Boer J., Mol L., Punt C.J.A., de Wilt J.H.W., Koopman M. (2021). Sixty-Day Mortality of Patients with Metastatic Colorectal Cancer Randomized to Systemic Treatment vs Primary Tumor Resection Followed by Systemic Treatment: The CAIRO4 Phase 3 Randomized Clinical Trial. JAMA Surg..

[19] Rahbari N.N., Biondo S., Feißt M., Bruckner T., Rossion I., Luntz S., Bork U., Büchler M.W., Folprecht G., Kieser M. (2022). Randomized clinical trial on resection of the primary tumor versus no resection prior to systemic therapy in patients with colon cancer and synchronous unresectable metastases. J. Clin. Oncol..

[20] Byrd D.R., Brookland R.K., Washington M.K., Gershenwald J.E., Compton C., Hess K.R., Sullivan D.C., Jessup J.M., Madera M., Meyer L.R. (2017). AJCC Cancer Staging Manual.

[21] Oken M.M., Creech R.H., Tormey D.C., Horton J., Davis T.E., McFadden E.T., Carbone P.P. (1982). Toxicity and response criteria of the Eastern Cooperative Oncology Group. Am. J. Clin. Oncol..

[22] Rubin D.B., Thomas N. (1996). Matching using estimated propensity scores: Relating theory to practice. Biometrics.

[23] Sanford N.N., Folkert M.R., Aguilera T.A., Beg M.S., Kazmi S.A., Sanjeevaiah A., Zeh H.J., Farkas L. (2020). Trends in Primary Surgical Resection and Chemotherapy for Metastatic Colorectal Cancer, 2000–2016. Am. J. Clin. Oncol..

[24] Xu H., Xia Z., Jia X., Chen K., Li D., Dai Y., Tao M., Mao Y. (2015). Primary Tumor Resection Is Associated with Improved Survival in Stage IV Colorectal Cancer: An Instrumental Variable Analysis. Sci. Rep..

[25] Yun J.A., Huh J.W., Park Y.A., Cho Y.B., Yun S.H., Kim H.C., Lee W.Y., Chun H.K. (2014). The role of palliative resection for asymptomatic primary tumor in patients with unresectable stage IV colorectal cancer. Dis. Colon. Rectum.

[26] Wang Z., Liang L., Yu Y., Wang Y., Zhuang R., Chen Y., Cui Y., Zhou Y., Liu T. (2016). Primary Tumour Resection Could Improve the Survival of Unresectable Metastatic Colorectal Cancer Patients Receiving Bevacizumab-Containing Chemotherapy. Cell Physiol. Biochem..

[27] Watanabe A., Yamazaki K., Kinugasa Y., Tsukamoto S., Yamaguchi T., Shiomi A., Tsushima T., Yokota T., Todaka A., Machida N. (2014). Influence of primary tumor resection on survival in asymptomatic patients with incurable stage IV colorectal cancer. Int. J. Clin. Oncol..

[28] Poultsides G.A., Servais E.L., Saltz L.B., Patil S., Kemeny N.E., Guillem J.G., Weiser M., Temple L.K., Wong W.D., Paty P.B. (2009). Outcome of primary tumor in patients with synchronous stage IV colorectal cancer receiving combination chemotherapy without surgery as initial treatment. J. Clin. Oncol..

[29] Cetin B., Kaplan M.A., Berk V., Tufan G., Benekli M., Isikdogan A., Ozkan M., Coskun U., Buyukberber S. (2013). Bevacizumab-containing chemotherapy is safe in patients with unresectable metastatic colorectal cancer and a synchronous asymptomatic primary tumor. Jpn. J. Clin. Oncol..

[30] Kim M.S., Chung M., Ahn J.B., Kim C.W., Cho M.S., Shin S.J., Baek S.J., Hur H., Min B.S., Baik S.H. (2014). Clinical significance of primary tumor resection in colorectal cancer patients with synchronous unresectable metastasis. J. Surg. Oncol..

[31] Tebbutt N.C., Norman A.R., Cunningham D., Hill M.E., Tait D., Oates J., Livingston S., Andreyev J. (2003). Intestinal complications after chemotherapy for patients with unresected primary colorectal cancer and synchronous metastases. Gut.

[32] Lee B., Wong H.-L., Tacey M., Tie J., Wong R., Lee M., Nott L., Shapiro J., Jennens R., Turner N. (2017). The impact of bevacizumab in metastatic colorectal cancer with an intact primary tumor: Results from a large prospective cohort study. Asia Pac. J. Clin. Oncol..

[33] Korkmaz L., Coşkun H., Dane F., Karabulut B., Karaağaç M., Çabuk D., Karabulut S., Aykan N.F., Doruk H., Avcı N. (2018). Kras-mutation influences outcomes for palliative primary tumor resection in advanced colorectal cancer-a Turkish Oncology Group study. Surg. Oncol..

[34] Shida D., Hamaguchi T., Ochiai H., Tsukamoto S., Takashima A., Boku N., Kanemitsu Y. (2016). Prognostic Impact of Palliative Primary Tumor Resection for Unresectable Stage 4 Colorectal Cancer: Using a Propensity Score Analysis. Ann. Surg. Oncol..

[35] Kawamura H., Ogawa Y., Yamazaki H., Honda M., Kono K., Konno S., Fukuhara S., Yamamoto Y. (2021). Impact of Primary Tumor Resection on Mortality in Patients with Stage IV Colorectal Cancer with Unresectable Metastases: A Multicenter Retrospective Cohort Study. World J. Surg..

[36] Ferrand F., Malka D., Bourredjem A., Allonier C., Bouché O., Louafi S., Boige V., Mousseau M., Raoul J.L., Bedenne L. (2013). Impact of primary tumour resection on survival of patients with colorectal cancer and synchronous metastases treated by chemotherapy: Results from the multicenter, randomised trial Fédération Francophone de Cancérologie Digestive 9601. Eur. J. Cancer.

[37] Ruo L., Gougoutas C., Paty P.B., Guillem J.G., Cohen A.M., Wong W.D. (2003). Elective bowel resection for incurable stage IV colorectal cancer: Prognostic variables for asymptomatic patients. J. Am. Coll. Surg..

[38] Faron M., Pignon J.P., Malka D., Bourredjem A., Douillard J.Y., Adenis A., Elias D., Bouché O., Ducreux M. (2015). Is primary tumour resection associated with survival improvement in patients with colorectal cancer and unresectable synchronous metastases? A pooled analysis of individual data from four randomised trials. Eur. J. Cancer.

[39] Gresham G., Renouf D.J., Chan M., Kennecke H.F., Lim H.J., Brown C., Cheung W.Y. (2014). Association between palliative resection of the primary tumor and overall survival in a population-based cohort of metastatic colorectal cancer patients. Ann. Surg. Oncol..

[40] Ishihara S., Hayama T., Yamada H., Nozawa K., Matsuda K., Miyata H., Yoneyama S., Tanaka T., Tanaka J., Kiyomatsu T. (2014). Prognostic impact of primary tumor resection and lymph node dissection in stage IV colorectal cancer with unresectable metastasis: A propensity score analysis in a multicenter retrospective study. Ann. Surg. Oncol..

[41] Park J.H., Kim T.Y., Lee K.H., Han S.W., Oh D.Y., Im S.A., Kang G.H., Chie E.K., Ha S.W., Jeong S.Y. (2013). The beneficial effect of palliative resection in metastatic colorectal cancer. Br. J. Cancer.

[42] Shida D., Boku N., Tanabe T., Yoshida T., Tsukamoto S., Takashima A., Kanemitsu Y. (2019). Primary Tumor Resection for Stage IV Colorectal Cancer in the Era of Targeted Chemotherapy. J. Gastrointest. Surg..

[43] Su Y.C., Wu C.C., Su C.C., Hsieh M.C., Cheng C.L., Kao Yang Y.H. (2022). Comparative Effectiveness of Bevacizumab versus Cetuximab in Metastatic Colorectal Cancer Patients without Primary Tumor Resection. Cancers.

[44] Alawadi Z., Phatak U.R., Hu C.Y., Bailey C.E., You Y.N., Kao L.S., Massarweh N.N., Feig B.W., Rodriguez-Bigas M.A., Skibber J.M. (2017). Comparative effectiveness of primary tumor resection in patients with stage IV colon cancer. Cancer.

[45] van Rooijen K.L., Shi Q., Goey K.K.H., Meyers J., Heinemann V., Diaz-Rubio E., Aranda E., Falcone A., Green E., de Gramont A. (2018). Prognostic value of primary tumour resection in synchronous metastatic colorectal cancer: Individual patient data analysis of first-line randomised trials from the ARCAD database. Eur. J. Cancer.

[46] Park E.J., Baek J.H., Choi G.S., Park W.C., Yu C.S., Kang S.B., Min B.S., Kim J.H., Kim H.R., Lee B.H. (2020). The Role of Primary Tumor Resection in Colorectal Cancer Patients with Asymptomatic, Synchronous, Unresectable Metastasis: A Multicenter Randomized Controlled Trial. Cancers.

[47] Ahmed S., Fields A., Pahwa P., Chandra-Kanthan S., Zaidi A., Le D., Haider K., Reeder B., Leis A. (2015). Surgical Resection of Primary Tumor in Asymptomatic or Minimally Symptomatic Patients with Stage IV Colorectal Cancer: A Canadian Province Experience. Clin. Colorectal. Cancer.

[48] Yoon Y.S., Kim C.W., Lim S.B., Yu C.S., Kim S.Y., Kim T.W., Kim M.J., Kim J.C. (2014). Palliative surgery in patients with unresectable colorectal liver metastases: A propensity score matching analysis. J. Surg. Oncol..

[49] Zhang R.X., Ma W.J., Gu Y.T., Zhang T.Q., Huang Z.M., Lu Z.H., Gu Y.K. (2017). Primary tumor location as a predictor of the benefit of palliative resection for colorectal cancer with unresectable metastasis. World J. Surg. Oncol..

[50] Kim J.H., Jin S., Jeon M.J., Jung H.Y., Byun S., Jung K., Kim S.E., Moon W., Park M.I., Park S.J. (2020). Survival Benefit of Palliative Primary Tumor Resection Based on Tumor Location in Patients with Metastatic Colorectal Cancer: A Single-center Retrospective Study. Korean J. Gastroenterol..

[51] Liang L., Tian J., Yu Y., Wang Z., Peng K., Liu R., Wang Y., Xu X., Li H., Zhuang R. (2018). An Analysis of Relationship Between RAS Mutations and Prognosis of Primary Tumour Resection for Metastatic Colorectal Cancer Patients. Cell Physiol. Biochem..

[52] Gulack B.C., Nussbaum D.P., Keenan J.E., Ganapathi A.M., Sun Z., Worni M., Migaly J., Mantyh C.R. (2016). Surgical Resection of the Primary Tumor in Stage IV Colorectal Cancer without Metastasectomy is Associated with Improved Overall Survival Compared with Chemotherapy/Radiation Therapy Alone. Dis. Colon. Rectum..

