# Supplementary material for: Clinical Outcomes of Upfront Primary Tumor Resection in Synchronous Unresectable Metastatic Colorectal Cancer
Source: Cancers (Basel). 2023 Oct 19;15(20):5057. doi: 10.3390/cancers15205057 (PMC10605032; doi:10.3390/cancers15205057)
Supplement: Supplementary file 1 [file cancers-15-05057-s001.zip › cancers-2588021-supplementary.pdf]

Table S1. Propensity score-matched pairs (n = 84)

Characteristic	Upfront PTR N = 42 (%)	Upfront chemotherapy n = 42 (%)	*p* value
ECOG performance status			
0/1	29 (69.0)	29 (69.0)	1.000
≥2	13 (31.0)	13 (31.0)	
Primary tumor location			
Right-sided	17 (40.5)	9 (21.4)	0.059
Left-sided	25 (59.5)	33 (78.6)	
Clinical T stage			
T3	13 (31.0)	13 (31.0)	1.000
T4	29 (69.0)	29 (69.0)	
Clinical M stage			
M1a	21(50.0)	25 (59.5)	0.618
M1b	6 (14.3)	6 (14.3)	
M1c	15 (35.7)	11 (26.2)	
No. of organ metastasis			
0 or 1	30 (71.4)	30 (71.4)	1.000
≥2	12 (28.6)	12 (28.6)	

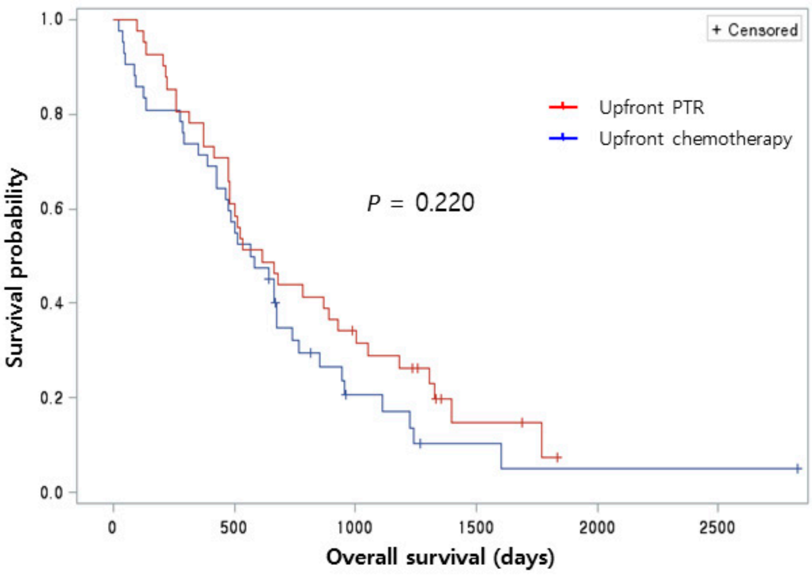

Figure S1. A Kaplan–Meier curve of overall survival in propensity matched population (n = 84). PTR, primary tumor resection.

Table S2. Summary of randomized trials comparing upfront PTR and CTx

Author	Study period	Symptom	No. of patients	CTx	Primary end point	HR (95% CI)	*P* value	Comment
Kanemitsu	June 2012 – September 2019	Asymptomatic	81 : PTR<br>84 : CTx	mFOLFOX/ CapeOx with bevacizumab	OS	1.10; 95% CI, 0.76 to 1.59	0.69	early termination of the trial because of futility
van der Kruijssen (CAIRO4)	August 2012– December 2019	Asymptomatic	97: PTR<br>99: CTx	fluoropyrimidine-based chemotherapy with bevacizumab	OS but 60-day mortality rates was reported in this paper	10% (95% CI, 5%-18%)<br>2% (95% CI, 1%-7%)	0.048	No report on OS
Rahbari, (SYNCHRONOUS trial/ CCRe-IV)	September 2011– March 2013	Asymptomatic	187: PTR<br>206: CTx	Various	OS	HR 0.95, 95% CI: 0.743-1.215	0.685	45 (24.1%) and 13 (6.4%) patients did not receive any chemotherapy in the PTR and CTX arm, respectively
Park	May 2013 – April 2016	Asymptomatic	26: PTR<br>22: CTx	Nor reported	2-year OS	69.5%<br>44.8%	0.058	ended early due to a lack of patient enrollment and cessation of funding

PTR, primary tumor resection; CTx, chemotherapy, HR, hazard ratio; CI, confidence interval; mFOLFOX, modified 5-FU/leucovorin/oxaliplatin; CapeOx, capecitabine/oxaliplatin; OS, overall survival
